# A Double-Switch Cell Fusion-Inducible Transgene Expression System for Neural Stem Cell-Based Antiglioma Gene Therapy

**DOI:** 10.1155/2015/649080

**Published:** 2015-05-05

**Authors:** Yumei Luo, Detu Zhu, Dang Hoang Lam, Juan Huang, Yi Tang, Xitu Luo, Shu Wang

**Affiliations:** ^1^Key Laboratory for Major Obstetric Diseases of Guangdong Province, The Third Affiliated Hospital of Guangzhou Medical University, Guangzhou 510150, China; ^2^Department of Biological Sciences, National University of Singapore, Singapore 117543; ^3^Institute of Bioengineering and Nanotechnology, Singapore 138669; ^4^Department of General Surgery, The Third Affiliated Hospital of Guangzhou Medical University, Guangzhou 510150, China

## Abstract

Recent progress in neural stem cell- (NSC-) based tumor-targeted gene therapy showed that NSC vectors expressing an artificially engineered viral fusogenic protein, VSV-G H162R, could cause tumor cell death specifically under acidic tumor microenvironment by syncytia formation; however, the killing efficiency still had much room to improve. In the view that coexpression of another antitumoral gene with VSV-G can augment the bystander effect, a synthetic regulatory system that triggers transgene expression in a cell fusion-inducible manner has been proposed. Here we have developed a double-switch cell fusion-inducible transgene expression system (DoFIT) to drive transgene expression upon VSV-G-mediated NSC-glioma cell fusion. In this binary system, transgene expression is coregulated by a glioma-specific promoter and targeting sequences of a microRNA (miR) that is highly expressed in NSCs but lowly expressed in glioma cells. Thus, transgene expression is “switched off” by the miR in NSC vectors, but after cell fusion with glioma cells, the miR is diluted and loses its suppressive effect. Meanwhile, in the syncytia, transgene expression is “switched on” by the glioma-specific promoter. Our *in vitro* and *in vivo* experimental data show that DoFIT successfully abolishes luciferase reporter gene expression in NSC vectors but activates it specifically after VSV-G-mediated NSC-glioma cell fusion.

## 1. Introduction

Over the past decade significant progress has been made in development of neural stem cells (NSCs) as a novel gene delivery vector for antiglioma therapy [1]. NSCs are multipotent stem cells that give rise to the three fundamental neural lineages, neurons, astrocytes, and oligodendrocytes, throughout the central nervous system. When brain tumors are present, NSCs are capable of migrating through the brain parenchyma, by either existing or atypical routes, to home in on the tumor foci, including the original site and distant “satellite lesions” [1]. In animal models, the strong neoplastic tropism of NSCs has been extensively exploited for targeted delivery of therapeutic genes, such as the suicide gene thymidine kinase (TK), into brain tumors. These studies demonstrated that the genetically engineered NSC vectors can infiltrate the original tumor mass and chase down the advancing tumor satellites regardless of the injection location and its distance to the tumor and significantly suppress the tumor growth via overexpressing the therapeutic transgenes [2–6]. However, it was found that constant expression of the suicide gene TK from off-target NSCs caused significant cytotoxic effects on normal brain tissue [7]. Thus, it raises a safety concern that the use of these NSC vectors may deteriorate the situations of the patients.

To harness the cytotoxic effect of NSC vectors, we have previously engineered a novel antitumor gene, vesicular stomatitis virus G glycoprotein (VSV-G) H162R mutant, to eliminate tumor cells specifically under the acidic tumor microenvironment [8]. VSV-G is one class of the fusogenic membrane glycoprotein (FMG) gene that can exert strong bystander killing effect via induction of multinucleated syncytia among the tumor cells [9]. However, the fusogenic capacity of the tumor acidosis-targeted H162R mutant is weaker than the wild-type; thus its killing efficacy still has much room to improve. Previous studies have shown that coexpression of suicide genes [10, 11], oncolytic viruses [12, 13], antitumoral chemokines [14], and cytokines [15, 16] with FMG can augment the tumor killing effects. Hence, we reason that induction of another transgene, such as the suicide gene TK, in the NSC/glioma syncytia could be a means to enhance the therapeutic efficacy of VSV-G-expressing NSC vectors.

To realize this while avoiding off-target side effects, it is desirable to restrict the transgene expression in a cell fusion-inducible manner. Double switching is originally a safety engineering practice that closes or opens both the positive and negative sides of an electrical circuit to prevent shock hazard in electric devices, which is an example of using redundancy to increase safety. Two recent studies have applied similar strategy to synthesize double-switch cellular circuits that couple transcriptional targeting and microRNA (miR) regulation to achieve precise control of transgene expression [7, 17]. In both studies, tumor-targeting promoters were used to activate transgene expression under on-target conditions, while highly expressed endogenous miRs were employed to knock down transgene expression under off-target conditions. The data showed that these double-switch expression cassettes obtained significantly higher specificities than single-promoter expression cassettes as the off-target “promoter leakage” was diminished by the “miR blockage.”

Here we proposed a double-switch cell fusion-inducible transgene expression system (DoFIT) to enable therapeutic transgene induction upon VSV-G-mediated NSC/glioma syncytia formation. In this binary system, the transgene expression is coregulated by a glioma-specific promoter and targeting sequences of a miR that is highly expressed in NSCs but lowly expressed in glioma cells. Thus, the miR is employed to “switch off” the transgene expression in NSC vectors; however, after VSV-G-mediated cell fusion with glioma cells, the miR is diluted and its suppressive effect is gone. Meanwhile, in the syncytia, the glioma-specific promoter “switches on” the transgene expression. Our data showed that this combinatorial control using transcriptional targeting and miR regulation resulted in high transgene expression in glioma but negligible off-target expression in a mouse model.

## 2. Materials and Methods

### 2.1. Cell Culture

Human malignant glioma cell lines U87 and U251 were purchased from American Type Culture Collection (Manassas, VA) and maintained in Dulbecco's modified Eagle's medium (DMEM) supplemented with 10% fetal bovine serum (FBS), 2 mM L-glutamine, 50 U/mL penicillin, and 50 *μ*g/mL streptomycin.

NSCs were derived from human induced pluripotent stem cells (iPSCs) using an adherent monoculture differentiation method as described previously [18]. In brief, iPSC colonies were detached from the 6-well cell-culture plate 7 days after plating by mechanical cutting. Then, iPSCs were then dissociated using TrypLE Express Dissociation Enzyme (Invitrogen) and plated onto a 0.1% gelatin-coated 6-well cell-culture plate at a density of 10^6^ per well and cultured in NSC medium, which was a 1 : 1 mixture of DMEM/F12 (Invitrogen) supplemented with 2% B27 (Invitrogen), 2 mM L-glutamine, 50 U/mL penicillin, 50 *μ*g/mL streptomycin, 20 ng/mL EGF (Sigma-Aldrich), and 20 ng/mL bFGF (Invitrogen). Half of the cell-culture medium was changed every 2 days. After 7 days of differentiation, the cells reached 90% confluence and were split at ratio of 1 : 2. After 1 month of expansion, NSCs were derived from iPSCs. NSCs were digested using TrypLE for cell passage and subcultured at ratio of 1 : 2 twice weekly.

### 2.2. Plasmid Constructs

pGL4.11 (Promega) carrying the luc2P reporter gene was used as a starting backbone to construct double-switch transgene expression cassettes through multistep subcloning. Firstly, all promoters used in this study were placed upstream of the reporter gene. Secondly, 4 × microRNA targeting sequences (mirT) were designed to be perfectly complementary to the respective microRNA (in lowercase in Table 1) with 3 different linkers spacing each targeting sequence. The respective sense and antisense strands of the 4 × mirT oligonucleotides were phosphorylated, annealed, and then inserted downstream of the reporter gene. A control scramble targeting sequence (ScrT) of the same size was designed based on the lack of significant similarity to any known microRNA and subcloned into the same region (Table 1).

### 2.3. Luciferase Assay

Subconfluent cells in 48-well plate were transfected with plasmids encoding luciferase reporter gene at 400 ng per well, using 1.2 *μ*L Fugene 6 (Roche) according to the manufacturer's protocol. After 24 h, the cells were lysed by freeze-thaw method and the supernatants were measured for luciferase activity using Luciferase Assay System (Promega) according to the manufacturer's instructions. All samples were assayed in triplicate.

### 2.4. MicroRNA qPCR

Small RNA was isolated using PureLink MicroRNA Isolation Kit (Invitrogen) and treated with TURBO DNA-free DNase (Ambion). Poly(A) tailing and cDNA synthesis of the DNAse-treated small RNA were performed using Ncode VILO MicroRNA cDNA Synthesis Kit (Invitrogen) according to the manufacturer's protocol. The forward primers for qRT-PCR analysis were designed based on entire known mature microRNA sequence, with additional 3 “A”s at the 3′ end to improve amplification specificity (Table 2). The reverse primer used was the Universal Primer in the EXPRESS SYBR GreenER MicroRNA qRT-PCR Kit (Invitrogen). 5S rRNA was selected as the internal reference gene for PCR quantification. To determine absolute copy number, a standard curve was generated using a synthetic LIN-4 RNA oligonucleotide.

qPCR was performed on iQ5 RT-PCR Detection System (BioRad). All reactions were run in triplicate.

### 2.5. Animal Experiment

Five BALB/c mice were anesthetized (132 *μ*g/g ketamine and 8.8 *μ*g/g xylazine) and received stereotactically guided injections of 10^6^ U251 cells in 10 *μ*L PBS through a 30-gauge Hamilton syringe into the right forebrain to allow glioma formation. After 1 week, 10^6^ DoFIT-NSCs were injected into the left forebrain and glioma xenografts in the right forebrain, respectively. On the following 2 days,* in vivo* luciferase reporter gene expression levels were measured by the Xenogen IVIS-100 bioimaging system (Caliper).

### 2.6. Statistical Analysis

All data are represented as mean ± s.d. The statistical significance of differences was determined by Student's *t*-test or the two-factor analysis of variance analysis (ANOVA). A *P* value of <0.05 was considered to be statistically significant.

## 3. Results

### 3.1. Selection of a Glioma-Specific Promoter

**Figure 1:**
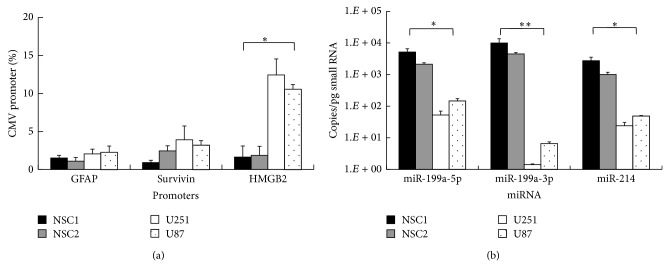
Selection of glioma-specific promoter and NSC-specific miR. (a) Promoter activities of the lineage-specific promoter GFAP, tumor-specific promoter Survivin, and glioma-specific promoter HMGB2 compared to the strong universal promoter CMV in different NSC and glioma cell lines are quantified by luciferase assays. (b) Absolute expression levels of miR-199a/214 cluster members miR-199a-5p, miR-199a-3p, and miR-214 in different NSC and glioma cell lines are quantified by qPCR. miR copy numbers were calculated based on a standard curve generated using a synthetic LIN-4 RNA oligonucleotide. Error bars: s.d. ^*^ *P* < 0.05, ^**^ *P* < 0.01.

Firstly, we wish to select a promoter which has high activity in glioma cells but low activity in NSCs. Based on literatures, the astroglial lineage-specific promoter GFAP [7], the tumor-specific promoter Survivin [19], and the glioma-specific promoter HMGB2 [20] were chosen as candidates. The promoter activities were tested by luciferase assay in iPSC-derived NSC lines NSC1 and NSC2 and glioma cell lines U251 and U87. And the results were normalized by the activity of the strong universal promoter CMV (Figure 1(a)). The results showed that the HMGB2 promoter had the highest activity in glioma cell lines among all promoters. However, its averaged activity difference between NSC and glioma cell lines was merely 3.9-fold, thus requiring endogenous miR as an additional barrier to avoid the potential “promoter leakage.”

### 3.2. Selection of an NSC-Specific miR

To select a miR which is expressed high in NSCs but low in glioma cells, we checked previous microRNA microarray data of the above NSC and glioma cell lines (unpublished data). As a result, hsa-miR-199a-5p, hsa-miR-199a-3p, and hsa-miR-214, all members of the miR-199a/214 cluster located on 1q24.3, were selected as candidates. miR qRT-PCR was employed to figure out their absolute expression levels in NSC1, NSC2, U87, and U251 (Figure 1(b)). The results showed that miR-199a-3p has the highest expression levels in NSC lines, up to 4.5 and 10 k copies per pg small RNA in NSC1 and NSC2, respectively. In addition, its specificity between NSC and glioma cell lines is also the highest among all candidate miRs, more than 682-fold. In both U87 and U251, its expression levels are much less than 100 copies per pg small RNA, which is insufficient to abolish transgene expression according to previous report [21]. Therefore, miR-199a-3p was chosen as the optimal suppressor of transgene expression within NSC vectors for glioma targeting.

### 3.3. Double-Switch Glioma-Specific Transgene Expression Cassette

**Figure 2:**
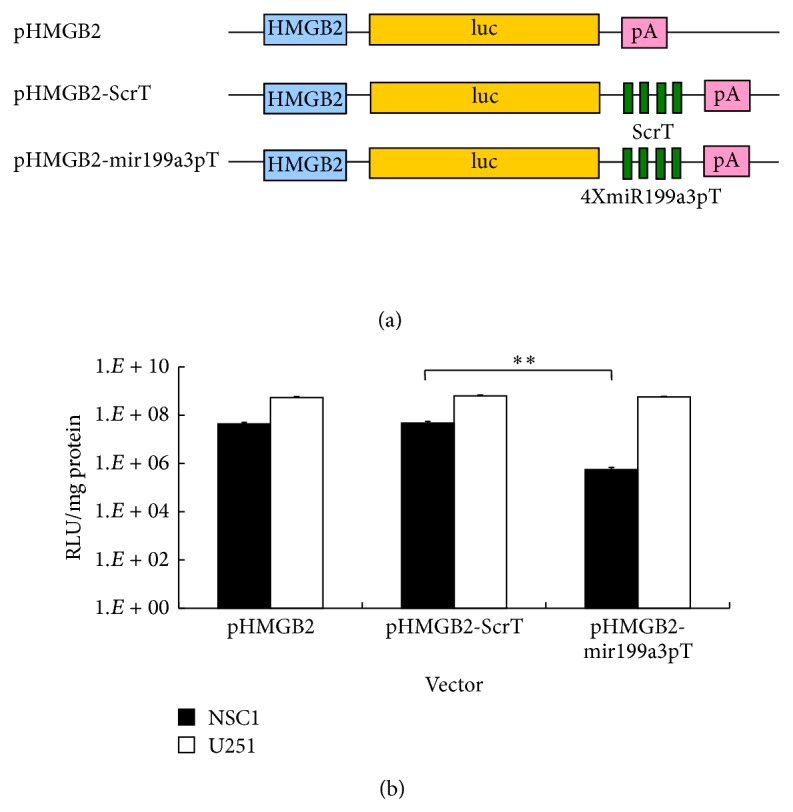
Combinatory effect of optHRP and miR-199a-5p on transgene regulation. (a) Schematic representation of the combinatorial expression cassettes containing the HMGB2 promoter and miRNA target sequences. HMGB2, high mobility group box 2 gene promoter; luc, luciferase reporter gene; miR-199a-3p and scramble target sequences as detailed in Table 1 were inserted into 3′-UTR, respectively; pA, polyA signal. (b) Transgene expression levels of different expression cassettes within NSC1 and U251 cell lines are quantified by luciferase assays. Error bars: s.d. ^*^ *P* < 0.05, ^**^ *P* < 0.01.

Glioma-specific transgene expression cassette under combinatory control of HMGB2 promoter and 4 × miR-199a-3p targeting sequences (pHMGB2-mir199a3pT) was constructed. Moreover, to rule out the possibility of less favorable transcription caused by introduction of a long repeat sequence into the 3′-UTR, a control construct pHMGB2-ScrT with a mismatched miR targeting sequence of the same length in the 3′-UTR was generated (Figure 2(a)). The original pHMGB2 construct and the above 2 new constructs were tested by luciferase assay in U251 and NSC1. The results indicated that, compared to pHMGB2-ScrT, pHMGB2-mir199a3pT obtains a reduction of luciferase activity up to 98.7% in NSC1. Additionally, the combinatorially regulated construct showed no significant decrease of transgene expression in U251 compared to other constructs (Figure 2(b)). Therefore, the double-switch construct pHMGB2-mir199a3pT was demonstrated to have a great inhibition on transgene expression by miR regulation in NSCs without compromising the promoter induction in glioma cells.

### 3.4. DoFIT Mediates Cell Fusion-Inducible Transgene Expression* In Vitro*

**Figure 3:**
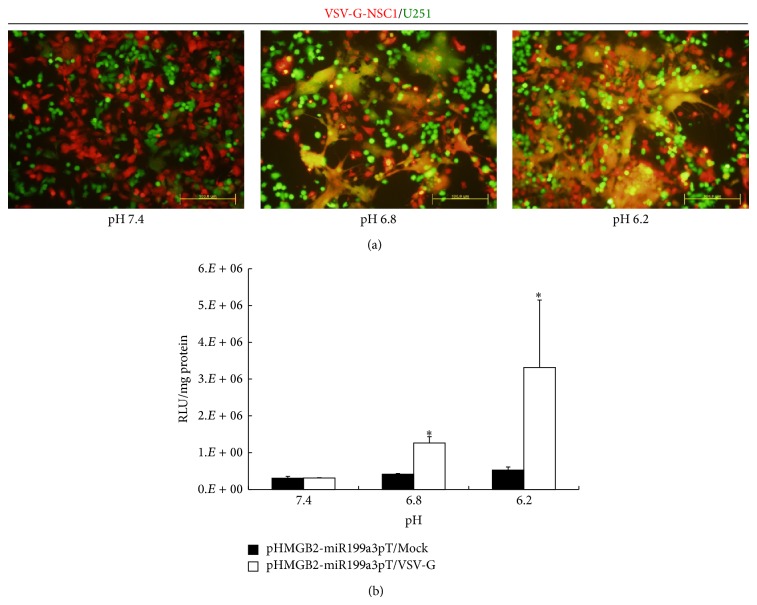
DoFIT mediates cell fusion-inducible transgene expression* in vitro*. (a) Low pH-dependent cell fusion between VSV-G-expressing NSC1 and U251 is examined by dual-color syncytia formation assays. (b) Transgene expression levels of cocultures between DoFIT-NSCs and U251 under different pH conditions are quantified by luciferase assays. Error bars: s.d. ^*^ *P* < 0.05, ^**^ *P* < 0.01.

Firstly, a dual-color syncytium formation assay was performed to test the ability of VSV-G to cause cell fusion between NSCs and glioma cells. NSC1 was transfected with pVSV-G, a plasmid encoding the VSV-G mutant H162R, and stained with the red-orange dye DiL and then cocultured with U251 at a ratio of 1 : 1 at pH 7.4, 6.8, and 6.2, respectively. pH 7.4 represents the normal physiological pH, pH 6.8 represents the typical acidic tumor extracellular pH (pH_e_), and pH 6.2 represents an optimal low pH that favors the fusogenic function of VSV-G. The results showed that VSV-G could mediate efficient cell fusion between NSCs and glioma cells at both pH 6.8 and 6.2 but not at the neutral pH 7.4 (Figure 3(a)).

Secondly, to examine whether pHMGB2-mir199a3pT can mediate cell fusion-inducible transgene expression* in vitro*, cell fusion luciferase assay was applied to NSC1/U251 coculture. NSC1 was transfected with pHMGB2-mir199a3pT together with pVSV-G or a Mock vector. Then the transfected cells were cocultured with U251 at a ratio of 1 : 1 under pH 7.4, 6.8, and 6.2, respectively. After 24 h, luciferase activity was measured. The results indicated that, at pH 7.4, no transgene expression was induced. At pH 6.8, only pHMGB2-mir199a3pT/VSV-G was induced. The induction ratio was 3-fold compared to pHMGB2-mir199a3pT/Mock. At pH 6.2, both pHMGB2-mir199a3pT/VSV-G and pHMGB2-mir199a3pT/Mock were induced (Figure 3(b)). Thus, it demonstrated that DoFIT can mediate tumor pH_e_-dependent cell fusion-inducible transgene expression* in vitro*.

### 3.5. DoFIT Mediates Glioma Site-Targeted Transgene Expression* In Vivo*

**Figure 4:**
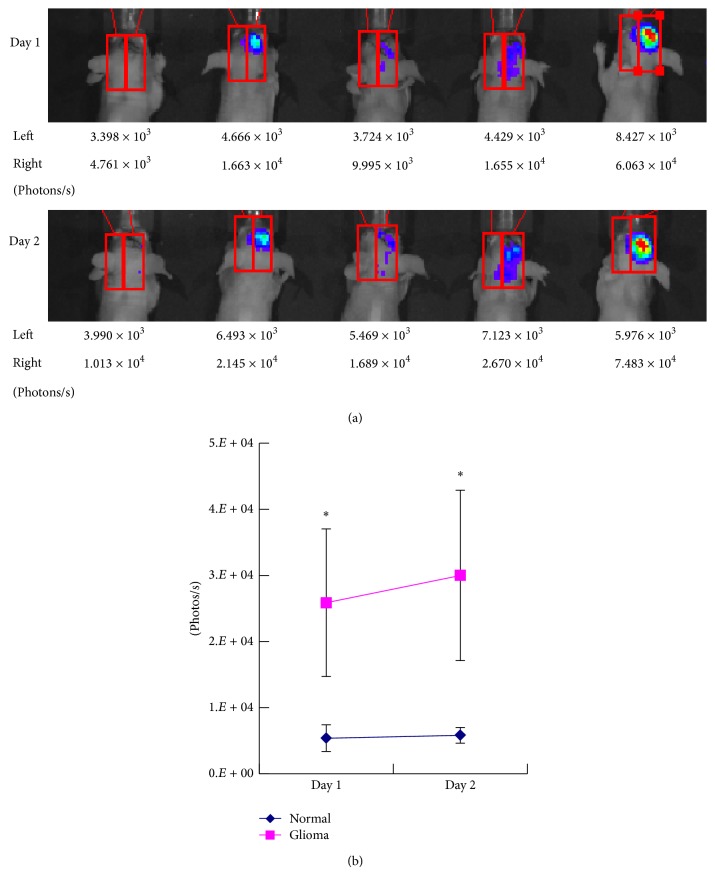
DoFIT mediates NSC-delivered glioma-specific transgene expression* in vivo*. (a) Bioluminescent images showing transgene expression in the glioma-bearing mice. U251 cells are inoculated into the right forebrains of the mice. After the gliomas develop, DoFIT-NSCs are inoculated into both the left and the right forebrains of the mice. Bioluminescent images are taken on day 1 and day 2 after NSC inoculation. The luminescent readings (photons/sec) of both left and right forebrains are indicated below the images. (b) Line graph showing trends of the averaged transgene expression levels in the glioma sites (right forebrain) and the normal sites (left forebrain) over day 1 and day 2 after NSC inoculation. Statistical significance of glioma sites versus normal sites at each time point was calculated by Student's *t*-test. Error bars: s.d. ^*^ *P* < 0.05.

To further examine whether DoFIT can mediate glioma-targeted transgene expression* in vivo*, an animal study was performed using an orthotopic mouse model of glioma. In brief, U251 was implanted into the right forebrain of BALB/c mice in advance to allow glioma formation. After 1 week, 10^6^ DoFIT-transfected NSCs were injected into the control left forebrains and glioma-xenografted right forebrains, respectively. On the following 2 days, luciferase reporter gene expression levels in the mice were measured using a live animal imaging platform. The results showed that DoFIT-NSCs could mediate transgene expression in the glioma-xenografted right forebrains but remained silenced in the control left forebrains (Figure 4(a)). Moreover, the average luminescent level in the right forebrains elevated by 20% from day 1 to day 2 (Figure 4(b)), which was probably due to more NSC arrival into the glioma sites and more cell fusion events.

## 4. Discussion

We have shown here a combinatorial transgene expression system DoFIT, which couples glioma-specific promoter HMGB2 and miR-199a-3p regulation to enable high level of transgene activation upon VSV-G-mediated NSC/glioma cell fusion but remains silenced in NSC vectors under off-target conditions.

Annually, there are approximately 189,000 new brain tumor cases diagnosed and 142,000 deaths documented worldwide, 80% of which are due to gliomas [22]. In spite of the relatively low incidence of glioma, the highly lethal nature of this cancer results in a median survival time of 15 months for patients with glioblastoma multiforme, grade IV glioma [23]. Despite surgical resection, combination radiation therapy, and chemotherapy, the cancer usually relapses due to extensive invasion of tumor cells into the normal brain parenchyma and acquisition of therapeutic resistance [24]. Hence, there is an urgent need to develop NSC-based antiglioma gene therapy that is able to target multiple invasive tumor foci intracranially, thus improving the therapeutic efficacy for this deadly disease.

The great potential of NSCs in cancer gene therapy highlights the importance of a robust, reliable source for the large scale, standardized production of human NSCs that meets the requirements of good clinical practice. The use of human iPSCs has provided an accessible and stable source to produce unlimited amounts of NSCs for cell-based therapies [4]. Also, iPSC-based therapeutic approaches bypass the sensitive ethical issue of human embryonic stem cells [25] and the safety concern of immune rejection by allogeneic transplantation. In this study, two iPSC-derived NSC lines were generated and could be genetically manipulated for the test of our system* in vitro* and* in vivo*.

Remarkably, our study has demonstrated that the intrinsic differences in endogenous miR expression patterns can be exploited to segregate the transgene expression between closely related cellular lineages. In this study, several glioma-specific promoters were tested. However, none of their specificities between NSC and glioma cell lines are sufficiently satisfactory, probably due to some similar characteristics shared between NSCs and glioma cells. Fortunately, substantial studies from different groups have demonstrated the feasibility of employing endogenous miRNA to inhibit transgene expression in a cell type-specific manner [7, 21, 26, 27]. Similarly, by incorporating targeting sequences of a highly expressed endogenous miR in NSCs into the transgene expression cassette, the selectivity between NSC and glioma cell lines increases by approximately 100-fold, indicating a strong capacity of endogenous miR regulation to minimize the potential off-target transgene expression caused by “promoter leakage.” Consistent with previous study, our data show that miR lower than 100 copies per pg small RNA has no significant suppressive effects on transgene expression [21].

More importantly, the animal experiment using an orthotopic brain tumor mice model demonstrated that our combinatorial system achieved very robust biphase transgene expression* in vivo*. The DoFIT-NSCs injected into normal forebrains displayed undetectable luciferase gene expression; meanwhile, vectors in glioma sites exhibited strong luminescent signals, and the signals even increased on the next day. This is reasonable as our previous studies showed that there would be more NSC vectors migrating into the tumor sites and more VSV-G-mediated cell fusion occurred [8]. Hence, it proves that our binary regulatory system is capable of abolishing off-target transgene expression without compromising the on-target expression* in vivo*.

## 5. Conclusion

In summary, our work demonstrates an inducible, nonleaky transgene expression system which functions within NSC-based gene delivery vectors for VSV-G-mediated NSC/glioma cell fusion-inducible transgene expression. Our data exhibit that it is able to trigger robust luciferase reporter gene expression upon NSC/glioma cell fusion at tumor pH_e_ *in vitro* and at glioma sites* in vivo*. Most importantly, a negligible transgene expression level in off-target region is observed, indicating the increased safety by applying an additional barrier of miR regulation. Further refinement of this system may lead to the development of optimal cell-based gene delivery vector to target malignant gliomas.

## Figures and Tables

**Table 1:** MicroRNA targeting (mirT) sequences.

mirT	Sequence
miR-199a-3p	
S1	5′-CTAGATAAtaaccaatgtgcagactactgtCGATtaaccaatgtgcagactactgt-3′
S2	5′-ACGCGTtaaccaatgtgcagactactgtTCACtaaccaatgtgcagactactgtGCATG-3′
AS1	5′-ACGCGTacagtagtctgcacattggttaATCGacagtagtctgcacattggttaTTAT-3′
AS2	5′-CacagtagtctgcacattggttaGTGAacagtagtctgcacattggtta-3′
ScrT	
S1	5′-CTAGAtaatttatgatctgcgcgtggagacgcccgattttatgatctgcgcgtggagacgcc-3′
S2	5′-acgcgttttatgatctgcgcgtggagacgcctcactttatgatctgcgcgtggagacgccGCATG-3′
AS1	5′-acgcgtggcgtctccacgcgcagatcataaaatcgggcgtctccacgcgcagatcataaattaT-3′
AS2	5′-Cggcgtctccacgcgcagatcataaagtgaggcgtctccacgcgcagatcataaa-3′

**Table 2:** MicroRNA qPCR primers.

MicroRNA	Primer sequence
hsa-miR-199a-5p	5′-CCCAGTGTTCAGACTACCTGTTCAAA-3′
hsa-miR-199a-3p	5′-ACAGTAGTCTGCACATTGGTTAAAA-3′
hsa-miR-214	5′-ACAGCAGGCACAGACAGGCAGTAAA-3′
